# Comparative Susceptibility of Pathogenic Methicillin-Resistant and Methicillin-Susceptible *Staphylococcus pseudintermedius* to Empirical Cotrimazole for Canine Pyoderma

**DOI:** 10.3390/life13051210

**Published:** 2023-05-18

**Authors:** Usasom Khongsri, Peerawit Chongrattanameteekul, Sineenart Chantarachart, Kornravee Photichai, Nittaya Chanayat, Thanya Varinrak, Raktham Mektrirat, Nuttawan Srifawattana

**Affiliations:** 1 Department of Veterinary Biosciences and Public Health, Faculty of Veterinary Medicine, Chiang Mai University, Chiang Mai 50100, Thailand; usasom.khongs@cmu.ac.th (U.K.); peerawit_ch@cmu.ac.th (P.C.); 2 Small Animal Hospital, Faculty of Veterinary Medicine, Chiang Mai University, Chiang Mai 50200, Thailand; sineenart.ch@cmu.ac.th; 3 Center of Veterinary Diagnosis and Technology Transfer, Faculty of Veterinary Medicine, Chiang Mai University, Chiang Mai 50100, Thailand; kornravee.p@cmu.ac.th (K.P.); nittaya.cha@cmu.ac.th (N.C.); thanya.var@cmu.ac.th (T.V.); 4 Epidemiological and Innovative Research Group for Infectious Diseases, Chiang Mai University, Chiang Mai 50200, Thailand; 5 Research Center for Pharmaceutical Nanotechnology, Chiang Mai University, Chiang Mai 50200, Thailand

**Keywords:** antimicrobial resistance, methicillin, pharmacodynamics, pyoderma, staphylococci, sulfamethoxazole, trimethoprim

## Abstract

The prevalence of methicillin-resistant *Staphylococcus pseudintermedius* (MRSP) that causes pyoderma has been gradually shifting, according to many surveillance studies, with annual changes. The empirical cotrimazole regimen remains interesting, but research on cotrimazole susceptibility to MRSP is limited. The objective of this study was to evaluate the susceptibility of cotrimazole to canine pyoderma MRSP isolates. Sixty isolates of *S. pseudintermedius* were identified as 16 MRSP and 44 methicillin-susceptible *S. pseudintermedius* (MSSP) using an oxacillin disk diffusion test and VITEK 2 system with VITEK GP card. Using the VITEK 2 system with a VITEK AST-GP81 card, the susceptibility rates of MRSP (15.00%) and MSSP (35.00%) to cotrimazole was observed. The median MIC of cotrimazole on MSSP (median, ≤10; IQR, 10–320) was lower than that of MRSP (median, ≥320; IQR, 10–320) (*p =* 0.5889, Mann-Whitney test). Percent attainment of PK/PD targets in MRSP (q 12 h, 43.75; q 8 h, 43.75) were lower than that of MSSP (q 12 h, 52.27; q 8 h, 52.27) (*p* = 0.7710). These findings show the moderately phenotypic cotrimazole susceptibilities of both MRSP and MSSP. Further study is required to develop clinical trials examining the use of cotrimazole in dogs with pyoderma.

## 1. Introduction

Canine pyoderma remains one of the most frequently diagnosed skin infections in dogs [1]. An opportunistic gram-positive Staphylococci is thought to be the primary cause of skin infections in companion animals [2]. *Staphylococcus pseudintermedius* represents the most prevalent cause of canine pyoderma [3]. When infections are not appropriately treated, *S. pseudintermedius* might become resistant [4,5]. Consequently, several staphylococci from dermatological canine patients frequently exhibit methicillin resistance [6,7,8]. The majority of methicillin resistant isolates develop multidrug resistance (MDR), which is defined as resistance to at least three antimicrobial classes, and severely restricts the therapeutic options available in clinical practice [9,10]. Pan-susceptible strains within any given *S. pseudintermedius* continue to occur but have become infrequent in clinical practice. The presence of MDR staphylococci, which veterinary practitioners are increasingly encountering, makes managing pyoderma more expensive and challenging [11]. Moreover, the increased occurrence of infections caused by MDR and methicillin-resistant *S. pseudintermedius* (MRSP) is a public health concern due to the risk of zoonotic diseases in pet owners [12,13,14,15]. In addition, the Infectious Diseases Society of America (IDSA) established recommendations on the treatment for human methicillin-resistant coagulase positive staphylococcal infections, including skin and soft tissue infections, bacteremia, endocarditis, bone and joint infections and infections of central nervous system [16].

Antimicrobial medication, whether topical, systemic, or both, is suggested in all but the mildest cases of canine pyoderma, according to recent evidence-based guidelines [17]. First generation cephalosporins, amoxicillin-clavulanate, clindamycin and potentiated sulfonamides-trimethoprim (or ormetoprim) are the first tier of systemic antimicrobial treatment options for superficial bacterial folliculitis in dogs [18]. Antibiotic therapy for suspected bacterial infections is frequently effective. However, recurrences have been connected to resistance to a range of commonly prescribed antimicrobial drugs including β-lactam antibiotics [19] and clindamycin [20]. 

Although newer antimicrobial agents have mostly superseded sulfonamides in the treatment of many infections, they remain effective and are the agents of choice in many cases [21]. The bacterial enzyme dihydropteroate synthase, which turns para-aminobenzoic acid into dihydropteroic acid, the direct precursor of folic acid, is inhibited by sulfonamides [22]. Diaminopyrimidines, in combination with sulfonamides, has a synergistic effect by specifically inhibiting microbial dihydrofolate reductase, the enzyme that converts dihydrofolate to tetrahydrofolate. The use of cotrimoxazole (sulfamethoxazole trimethoprim; SXT) for the management of many kinds of infected animals has been well documented. This medication is also relatively affordable and can be utilized to treat a wide range of infections [23]. One such antifolate, cotrimazole, is an efficient antibacterial agent used as a last option to treat infections caused by methicillin-resistant *S. aureus* (MRSA) [24,25]. Moreover, the community-acquired MRSA is common; oral cotrimazole is a treatment option for skin and soft tissue infections [26]. Many methicillin-resistant Staphylococci (MRS) strains have been approved for cotrimazole treatment in animals [27]. As a consequence, non-β-lactam antimicrobials have become increasingly important. 

The antibiograms of *S. aureus* have been well established in human medicine. Despite this, *S. pseudointermedius* has received relatively little study attention in veterinary medicine, particularly regarding isolates from canine pyoderma in Thailand. Methicillin-resistant Staphylococcus pseudintermedius is becoming more common. As a result of antimicrobial resistant bacteria, treating skin infections in dogs is getting increasingly difficult. The objectives of this study were to compare antimicrobial susceptibility and distributions of minimum inhibitory concentration (MIC) of cotrimazole in *S. pseudintermedius* isolated from canine pyoderma, with a special focus on the differences between the isolates of methicillin-resistant *S. pseudintermedius* (MRSP) and methicillin-susceptible *S. pseudintermedius* (MSSP). The pharmacokinetic/pharmacodynamic (PK/PD) parameter in predicting the efficacy of cotrimoxazole was also evaluated. These research findings help to better understand antimicrobials for use in canine pyoderma, as well as the develop evidence-based research in veterinary practice. The study will support efforts in the fight against the spread of antimicrobial resistance and the behavioral changes associated with cotrimazole usage.

## 2. Materials and Methods

### 2.1. Studied Specimen and Ethical Approval

This prospective study was conducted at the Center of veterinary diagnosis and technology transfer, Faculty of Veterinary Medicine, Chiang Mai University, Thailand. We tested and analyzed a total of sixty sequential, non-duplicate isolates collected from clinical specimens of dogs with pyoderma at a Small Animal Hospital, Faculty of Veterinary Medicine, Chiang Mai University, Thailand between January to December 2022. The research study was ethically approved by the Animal Care and Use Committee, Faculty of Veterinary Medicine, Chiang Mai University (approval number: S3/2563). The Institute Biosafety Committee, Chiang Mai University, also granted permission to test the pathogens (approval number: CMUIBC A-0763011).

### 2.2. Primary Identification of Coagulase-Positive Staphylococci

All bacterial samples were cultivated using Tryptic soy agar plates (Merck, Darmstadt, Germany) with 5% defibrinated sheep blood, and aerobically incubated at 37 °C for 24 h. The development of presumed staphylococcal colonies was determined by morphological properties including hemolytic characteristics, pigmentation, and typical colony shape. The suspected colonies were sub-cultured onto Mannitol salt agar (Oxoid Ltd., Basingstoke, UK) and incubated aerobically at 37 °C for 24 h. The purified colonies were morphologically identified by gram staining, catalase, oxidase, clumping factor, and tube coagulase tests. 

### 2.3. Antimicrobial Susceptibility Testing

Antimicrobial susceptibility testing (AST) was performed by the Kirby-Bauer disk diffusion method according to The Clinical and Laboratory Standards Institute (CLSI) standards. The coagulase-positive staphylococcal isolates were inoculated into Bacto™ Todd-Hewitt broth (THB) (Becton Dickinson & Co., Sparks, MD, USA) and incubated aerobically at 37 °C for 24 h. Bacterial culture was adjusted to a concentration of 1.5 × 10^8^ colony-forming units/mL using a McFarland densitometer (Grant Instruments, Cambridgeshire, UK) and swabbed on BBL™ Mueller-Hinton agar plates (Becton Dickinson & Co., Sparks, MD, USA). All isolates were subjected to the AST by the agar diffusion method with two antimicrobial disks including 1 µg oxacillin (OX) and 23.75 µg sulfamethoxazole-1.25 µg trimethoprim. After 24 h incubation at 30 °C, the size of the bacterial growth inhibition zone dimeters (ZD) was interpreted as sensitive (S) (≥16 mm) and resistant (R), including intermediate resistant (11–15 mm) and resistant (≤10 mm), according to the antimicrobial breakpoints for Staphylococci detailed in the CLSI guidelines. 

### 2.4. Confirmation of Staphylococcus pseudintermedius

The presumptive coagulase-positive staphylococcal isolates were then subjected to identification using the VITEK 2 Compact instrument (Biomerieux, Marcy l’Etoile, France) according to the manufacturer’s instructions. The disposable VITEK GP cards were used to identify *S. pseudintermedius.* The GP identity card is a completely closed system, requiring minimal additional reagents. The card was loaded onto a cassette made specifically for the VITEK 2 system, placed inside the apparatus, automatically placed in a vacuum, protected from the elements, and automatically subjected to colorimetric measurement every 15 min for a maximal incubating period of 8 h. The VITEK 2 database version 9.02 was applied. This database enables bacterial identification in a kinetic mode commencing 180 min after the initial beginning of the incubation process.

### 2.5. Determination of Minimum Inhibitory Concentration Values

The presumptive isolates of *S. pseudintermedius* were also subsequently investigated for antimicrobial susceptibility using the VITEK 2 Compact apparatus. The cefoxitin testing for MRSP confirmation and the MICs of cotrimazole among MRSP and MSSP were achieved by using a single disposable VITEK AST-GP81 card per isolate. The AST-GP81 card was automatically inoculated with a bacterial suspension generated in 0.45% sodium chloride solution at a spectrophotometric turbidity of 0.5. Inoculum preparation and card filling were usually separated by less than 30 min and put into the Vitek-2 apparatus for incubation and reading. The Advanced Expert System was used to provide categorical interpretations of the Vitek-2 AST data.

### 2.6. Target Attainment of Pharmacokinetic and Pharmacodynamics

The pharmacokinetic profile of oral cotrimazole at a dosage of 30 mg/kg for once-daily administration in dogs was utilized [28]. Plasma concentration of cotrimazole above the MIC throughout the whole dosing interval (T_0–24_ > MIC) was performed for achieving a specific PK/PD criterion. Successful target attainment in more than half of the total dosing intervals qualifies as acceptable overall for reaching the criterion. Probability of target attainment analysis is used to optimize dosing regimens of cotrimazole for patient dogs infected with MRSP and MSSP.

### 2.7. Statistical Analysis

Descriptive statistics were used to describe data, including frequency, percentage, proportion, range, average with standard deviation, and median with interquartile range to express the basic information on the tested bacteria. The distribution of ZD and MIC values of cotrimazole against bacterial isolates was plotted. The normality of the continuous variable distribution was examined using the Shipiro-Wilk test and normal Q-Q plots. The Mann-Whitney U test was used to assess differences between two independent groups when the dependent variable is ordinal or continuous but not normally distributed. In order to fit the data in the contingency table, the agreement of ZD results of tested isolates against oxacillin and cotrimazole were compared by Cohen’s kappa statistic. The oxacillin and cotrimazole susceptibility were also compared using odd ratios (ORs). Cohen’s kappa and OR statistics were also used to compare the MIC values of tested isolates against cefoxitin and cotrimazole. The median and 90th percentile were also calculated by regression equations. The association between two continuous variables of antimicrobial ZD and MIC values was assessed using Pearson’s correlation coefficients. Percentage probabilities of PK/PD target attainment of cotrimazole in the MRSP and MSSP groups were calculated by time-above-MIC targets. Chi-square tests were used to determine the association between two categorical variables and the difference in proportions. The differences between variables were considered statistically significant when the bicaudal probability was lower than 5% (*p* < 0.05) due to chance (error type I). Statistical analysis was performed with R statistical software (RStudio, Boston, MA, USA). 

## 3. Results 

### 3.1. Cotrimazole Susceptibility among Coagulase-Positive Staphylococci

**Figure 1:**
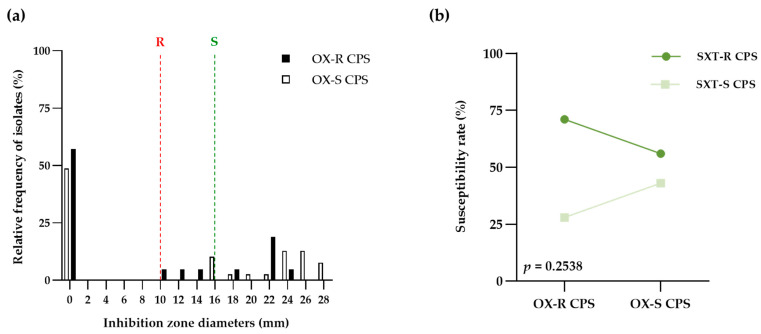
Comparison of inhibition zone diameter of cotrimazole between oxacillin-resistant coagulase-positive Staphylococci (OX-R CPS) and oxacillin-susceptible Staphylococci (OX-S CPS). (**a**) Bar graph compared the distributions of cotrimazole’s inhibition zone diameters between OX-R CPS (black bar) and OX-S CPS (white bar). Susceptible (green dash line) and resistant (red dash line) breakpoints recommended from CLSI guidelines (**b**) The point and connecting line graph showed the proportion of resistant (SXT-R CPS; dark green) and susceptible (SXT-S CPS; light green) to cotrimazole in OX-R CPS and OX-S CPS groups. The chi-square was used for statistical analysis and calculating *p*-values.

Sixty sequential isolates from patient dogs with pyoderma were identified as coagulase-positive staphylococci (CPS) by the conventional method according to CLSI guidelines. Data from the general comparison of the disk diffusion testing results for oxacillin and cotrimazole against all canine pyoderma CPS were demonstrated in Table 1. Among the tested isolates, the percentage of susceptibility to antimicrobials was as follows: 65.00% to oxacillin and 38.33% to cotrimazole [10.00% in oxacillin-resistant isolates (OX-R CPS) and 28.33% in oxacillin-susceptible isolates (OX-S CPS)]. Moreover, the cotrimazole susceptibility was related with a significant increase in chances for OX-S CPS isolates [odd ratio (OR), 1.90; 95% confidence interval (CI), 0.65–6.47] compared to OX-R CPS isolates (*p* = 0.2538). However, slight agreement between both antimicrobial agents was detected in 32 isolates (53.33% of the observations) with kappa coefficients 0.13 [95%CI, −0.09–0.34; standard error (SE) of kappa 0.11]. By the point of interception, the distribution of cotrimazole ZD on CPS isolates was described in Table 2 and Figure 1a. The ZD range (0–28 mm) and mean ± SD (10.15 ± 11.13) of cotrimazole among total CPS isolates was found. Interestingly, the median ZD of cotrimazole on OX-R CPS [median, 0; interquartile range (IQR), 0–19] was narrower than that of OX-S CPS (median, 15; IQR, 0–24) (*p =* 0.1631, Mann-Whitney test). Moreover, both OX-R CPS and OX-S CPS groups exhibited susceptibility to cotrimazole; they were 28.57% and 56.41, respectively (Figure 1b).

### 3.2. Cotrimazole Susceptibility among Pathogenic S. pseudintermedius

**Figure 2:**
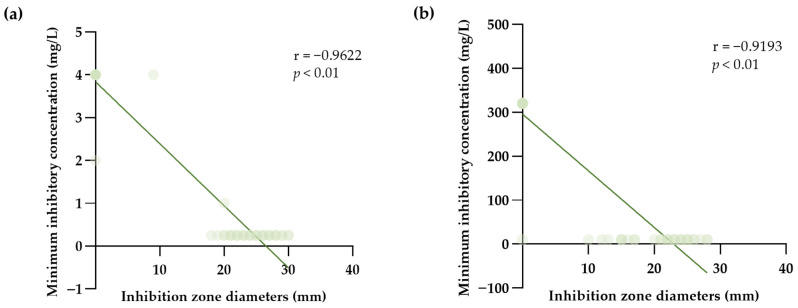
The correlation of MIC values and the inhibition zone diameters against 60 isolates of *S. pseudintermedius*. (**a**) Scatter diagram of oxacillin MIC and inhibition zone diameter with a data trend line. (**b**) Scatter diagram of cotrimazole MIC and inhibition zone diameter with a data trend line. The Pearson correlation was used for statistical analysis.

Sixty presumptive CPS isolates from dogs with pyoderma were confirmed as *S. pseudintermedius* by the VITEK 2 Compact instrument with VITEK GP cards. For cefoxitin testing, sixteen MRSP isolates were confirmed, whereas the remaining 44 MSSP isolates were identified. The MICs of cotrimazole in MRSP and MSSP were also achieved by using the VITEK AST-GP81 cards. The MICs and ZDs of oxacillin against tested *S. pseudintermedius* were well-correlated (correlation coefficient −0.9622, *p* < 0.01). The significant negative association for the MICs and ZDs of cotrimazole on *S. pseudintermedius* with correlation coefficient −0.9193 was also observed (*p* < 0.01) (Figure 2). 

**Figure 3:**
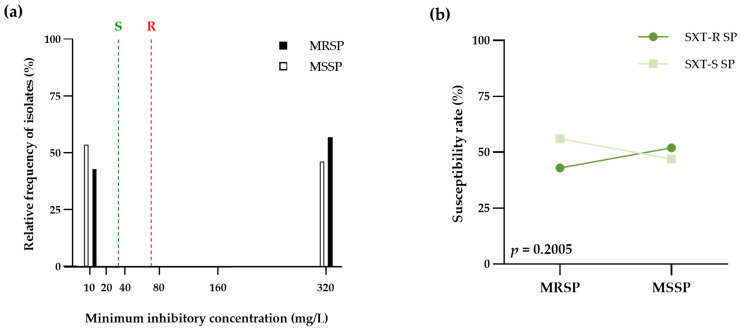
Comparison of minimum inhibitory concentration of cotrimazole between methicillin-resistant *S. pseudintermedius* (MRSP) and methicillin-susceptible *S. pseudintermedius* (MSSP). (**a**) Bar graph compared the distributions of cotrimazole’s minimum inhibitory concentration between MRSP (black bar) and MSSP (white bar). Susceptible (green dash line) and resistant (red dash line) breakpoints recommended by CLSI guidelines (**b**) The point and connecting line graph showed the proportion of resistant (SXT-R SP; dark green) and susceptible (SXT-S SP; light green) to cotrimazole in MRSP and MSSP groups. The chi-square was used for statistical analysis and calculating *p*-values.

The general information gleaned from the comparison of the MIC values for oxacillin and cotrimazole against sixty canine pyoderma *S. pseudintermedius* isolates was demonstrated in Table 3. The proportion of antimicrobial susceptibility among the tested isolates was 73.33% to cefoxitin surrogate marker and 50.00% to cotrimazole (15.00% in MRSP and 35.00% in MSSP). Moreover, the cotrimazole susceptibility showed a significant increase in chances for MSSP isolates (OR, 0.71; 95%CI, 0.23–2.40) compared to MRSP isolates (*p* = 0.5593). Interestingly, the susceptible rate of cotrimazole in the MRSP group was 56.25% (Figure 3b). In addition, the fair agreement between both antimicrobial agents was 28 isolates (46.67% of the observations) with kappa coefficients −0.067 (95%CI, −0.290–0.157; SE of kappa 0.11). The distribution of cotrimazole MIC on *S. pseudintermedius* isolates was described in Table 4 and Figure 3a. The median MIC of cotrimazole on MSSP (median, ≤10; IQR, 10–320) was lower than that of MRSP (median, ≥320; IQR, 10–320) (*p =* 0.5889, Mann-Whitney test). Moreover, the MIC_90_: MIC_50_ ratio of cotrimazole on MSSP (320/10) was also significantly lower than that of MRSP (320/320).

### 3.3. PK-PD Target Achieving of Cotrimazole on Pathogenic S. pseudintermedius

Cotrimazole achievement rates relating to *S. pseudintermedius* were evaluated based on the time the free drug stayed over the plasma MIC between treatment intervals (Table 5). The PK parameters of cotrimazole in dogs orally administered (30 mg/kg/24 h) were used. The result demonstrated that percentages of time above MIC on 24-, 12- and 8-h dosing intervals were 33.33, 66.66 and 100.00, respectively. With a 24-h dosing interval, both the MRSP and the MSSP had zero percentage probabilities of achieving the PK/PD target. Percent attainment of PK/PD targets in MRSP (q 12 h, 43.75; q 8 h, 43.75) were lower than that of MSSP (q 12 h, 52.27; q 8 h, 52.27) (*p* = 0.7710).

## 4. Discussion

The genus Staphylococcus is common in epidermal and nasal flora and leads to opportunity-based infections in both humans and animals. When compared to coagulase-negative staphylococci, CPS may result in more severe infections [29,30]. A great many prior investigations have demonstrated that *S. pseudintermedius* is a prevalent cause of canine pyoderma [29,31,32]. In a recent investigation, all examined isolates were conventionally identified as CPS. All CPS-tested isolates were indeed subsequently confirmed as *S. pseudintermedius* using the VITEK 2 Compact instrument and VITEK GP cards. This actively demonstrates that all pathogenic bacteria from clinical specimens of dogs with pyoderma were consistent with the previous studies. The range of concentrations determined by the Vitek-2 AST-GP card is in accordance with veterinary breakpoints established according to the current CLSI guidelines for the oxacillin utilized in this investigation [33].

The particular reason for using the VITEK 2 GP test technique was validated in accordance with the Association of Official Analytical Collaboration (AOAC) recommendations for the identification of Staphylococcus species usingthe Performance Tested Method^SM^ (PTM) and Official Methods of Analysis^SM^ (OMA). Furthermore, a previous research investigation indicated that the Vitek 2 Compact instrument accurately identifies 123 (93.2%; *n* = 132) different Staphylococcus strains from the database species. In addition, the research points out the remarkable accomplishment of the colorimetric Vitek 2 GP card, which is capable of being utilized in medical, veterinary, agricultural, and food laboratories [34]. As a consequence of this, previous research has recommended that the VITEK 2 GP test card method be used as the Official First Action for the detection of certain Gram-positive bacteria [35]. Moreover, the accuracy of VITEK-2 for phenotypic identification of Staphylococci is higher than that of the MicroScan and Crystal GP systems [36]. Through the use of molecular techniques, genotypic bacterial species might be reliably detected. Unfortunately, 16s rDNA sequencing did not confirm all bacterial isolates in this study.

The susceptibility of all CPS Isolates to cotrimazole was determined by AST using an OX disk. In accordance with EUCAST recommendations, oxacillin has been used as a surrogate for methicillin-resistant AST. The oxacillin disk (1 g) accurately predicted *mecA*-mediated methicillin resistance in *S. pseudintermedius.* [37,38]. Likewise, this study revealed that one-third of tested CPS (21 isolates) were OX-R CPS. Furthermore, 16 MRSP isolates were identified by the cefoxitin testing on OX-R CPS isolates using VITEK AST-GP81 cards. Previous investigations have demonstrated the correlation of cefoxitin and oxacillin ZD and MIC values with *mecA*/*mecC* PCR and the diagnostic test accuracy of disk diffusion relative to broth microdilution for canine clinical *S. pseudintermedius* [39]. Moreover, the Vitek 2 automated system also provides a highly reliable approach for detecting Staphylococcal *mecA* and *mecC* positive isolates [40,41,42]. This actively demonstrates that the five remaining isolates that showed oxacillin resistance but not the cefoxitin resistance phenotype, were classified as MSSP. However, 1.1% of methicillin-resistant Staphylococci probably remain sensitive to cefoxitin, according to a prior study [43]. 

In this study, the cotrimazole susceptibility among the tested *S. pseudintermedius* was 50.00% with the 50th percentile of the MIC less than 10 mg/L. The finding is consistent with previous research on the susceptibility of *S. pseudintermedius* isolated from infected dogs to cotrimazole [44,45,46]. Moreover, many previous studies demonstrated that the median MIC was less than 10 mg/L [47,48,49]. Surprisingly, this research also demonstrated that the cotrimazole sensitivity of the OX-R CPS and MRSP groups was 28.57% and 56.25%, respectively. These findings differed from the previous study in that MRSP was completely resistant to cotrimazole [50]. However, the MIC_50_/MIC_90_ ratio of cotrimazole on MSSP was much lower than that of MRSP. In addition, cotrimazole demonstrated a low MIC range for MRSP, and both MIC_90_ and MIC_50_ of MRSP were above the break point. These results suggest that MRSP developed resistance to cotrimazole at a higher rate than MSSP. We are aware of some limitations in our study. MIC values were determined using automated testing procedures rather than broth microdilution. The methicillin-resistance of bacterial strains is caused by the *mec* gene, which encodes a second penicillin binding protein with a low affinity for all β-lactam antibiotics [51]. Consequently, a group of antimetabolite sulfonamide has been less affected by *mec* gene mutation. However, Staphylococcus isolates may mutate a *DfrB* gene causing bacteria resistance if they are exposed to cotrimazole for a duration longer than two weeks [52].

Disk diffusion and broth microdilution methods have been approved as AST reference procedures by the CLSI and the EUCAST. The automated approaches are preferred to manual labor methods. The Vitek-2 system has been attached to the Advanced Expert System, which was developed to evaluate Vitek-2 results on the basis of specific bacterial species. AST reliability of Vitek-2 has been validated for numerous bacterial species of Staphylococci and Enterococci [53]. However, when testing antimicrobial agents against particular staphylococci, Vitek-2 might provide the results that are not accurate [54]. However, this present study revealed a strong correlation between ZD and MIC values of both oxacillin and cotrimazole against *S. pseudintermedius*; hence, ZD might be used to predict MIC values. These findings are in agreement with previously published research that reported almost perfect agreement between disk diffusion to Vitek-2 of oxacillin and cotrimazole against *S. pseudintermedius* isolates collected in clinical microbiology laboratories in Italy during 2018 to 2020 [55].

For surface and superficial conditions, topical therapy should be the only on-animal antimicrobial treatment administered. However, systemic treatment is suggested for deep pyoderma, multifocal or severe superficial infections in dogs that are not susceptible to topical therapy. This recommendation, which was reached through consensus among the Antimicrobial Guidelines Working Group of the International Society for Companion Animal Infectious Disease (ISCAID), focuses on the treatment of superficial bacterial folliculitis in particular and classifies antimicrobial agents into first- and second-tier categories [56]. In addition, the MIC value is necessary for antimicrobial treatment regimens that predict outcomes. Interestingly, this PK/PD target of cotrimazole might be reached with approximately 50% of both MRSP and MSSP. However, the pharmacodynamic parameter for optimizing the dose of cotrimazole is limited [57]. Cotrimazole has time-dependent bacteriostatic activity and the potential for concentration-dependent bactericidal activity against particular species [58]. The pharmacokinetic of cotrimazole in dogs has been also infrequently documented. As a result, achievement rates of cotrimazole to *S. pseudintermedius* were estimated based on the duration that the free drug remained over the plasma MIC between dosing intervals. The effectiveness of systemic antimicrobial medication for both MRSP and MSSP infections is primarily influenced by bacterial susceptibility. Moreover, it also depends on other criteria of rational drug use, including drug administration, correct dosing, and dog’s owner compliance [59]. Systemic therapy combined with topical antibiotic treatment is advised wherever possible to minimize contamination of the environmental microorganism and the likelihood of spreading the infection to other hosts, as well as to potentially decrease the total duration of systemic prescription.

Multi-center surveillance studies showed progressive shifts with annual trends of antimicrobial susceptibility [60]. The development of evidence-based research in veterinary medicine is essential since occurrences of pyoderma-causing MRSP have been reported [61,62]. Empirical antimicrobial agent selection is no longer reliable for pyoderma therapy in locations with high MRSP prevalence. The empirical amoxicillin-clavulanate and first-generation cephalosporins have a poor effectiveness for superficial bacterial folliculitis in dogs. Therefore, the treatment of canine pyoderma is becoming more challenging due to bacteria like MRSP. The outcomes showed that the remaining empirical cotrimazole regimen is an attractive alternative for the MRSP canine pyoderma. Future susceptibility tests should focus on *S. pseudintermedius* isolated from different geographic regions, time dynamics, and with an increased variety of antimicrobials susceptibility patterns.

## 5. Conclusions

The results highlighted the first study on cotrimazole susceptibility and PK/PD target attainment in pathogenic *S. pseudintermedius* isolated from dogs with pyoderma. Through this research, different levels of phenotypic cotrimazole susceptibility of MRSP and MSSP was observed. Interestingly, when given cotrimazole at at least a 12-h dosing interval, the attainment rate of pathogenic MRSP was more than 50%. Further studies are necessary to expand the clinical trial of cotrimazole in dogs with pyoderma. 

## Figures and Tables

**Table 1:** Comparison of phenotypic susceptibility for cotrimazole of oxacillin-resistant and susceptible Coagulase-Positive Staphylococci.

	SXT-R CPS	SXT-S CPS	Total
OX-R CPS	15	6	21
OX-S CPS	22	17	39
Total	37	23	60

OX-R CPS; oxacillin-resistant coagulase-positive Staphylococci. OX-S CPS; oxacillin-susceptible coagulase-positive Staphylococci. SXT-R CPS; cotrimazole-resistant coagulase-positive Staphylococci. SXT-S CPS; cotrimazole-susceptible coagulase-positive Staphylococci.

**Table 2:** Descriptive statistics of inhibition zone diameter (mm) using cotrimazole disk diffusion against 21 oxacillin-resistant and 39 susceptible coagulase-positive Staphylococci isolates.

Susceptibility	Pathogenic Bacteria		
	OX-R CPS	OX-S CPS	Total
Range	0–24	0–28	0–28
Mean ± SD	7.71 ^ns^ ± 9.69	11.46 ^ns^ ± 11.75	10.15 ± 11.13
Median (IQR)	0 ^ns^ (0–19)	15 ^ns^ (0–24)	0 (0–22)

OX-R CPS; oxacillin-resistant coagulase-positive Staphylococci. OX-S CPS; oxacillin-susceptible coagulase-positive Staphylococci. ^ns^; Not Significant (Mann-Whitney test; *p* > 0.05).

**Table 3:** Comparison of phenotypic susceptibility for cotrimazole of methicillin-resistant and susceptible *S. pseudintermedius*.

	SXT-R SP	SXT-S SP	Total
MRSP	7	9	16
MSSP	23	21	44
Total	30	30	60

MRSP; Methicillin-resistant *S. pseudintermedius*. MSSP; Methicillin-susceptible *S. pseudintermedius*. SXT-R SP; Cotrimazole-resistant *S. pseudintermedius*. SXT-S SP; Cotrimazole-susceptible *S. pseudintermedius*.

**Table 4:** Descriptive statistics of minimum inhibitory concentration (mg/L) of cotrimazole against 16 methicillin-resistant and 44 susceptible *S. pseudintermedius* using the VITEK 2 Compact instrument with VITEK AST-GP81 cards.

Susceptibility	Pathogenic Bacteria		
	MRSP	MSSP	Total
Median (IQR)	≥320 ^ns^ (10–320)	≤10 ^ns^ (10–320)	≤10 (10–320)
50th percentile	≥320	≤10	≤10
90th percentile	≥320	≥320	≥320
MIC_90_: MIC_50_	1	32	32

MRSP; Methicillin-resistant *S. pseudintermedius*. MSSP; Methicillin-susceptible *S. pseudintermedius*. ^ns^ = Not Significant.

**Table 5:** Percentage probabilities of pharmacokinetic and pharmacodynamics target attainment of oral cotrimazole with reference dose 30 mg/kg for each frequency regimens based on the evaluation of plasma concentration time above MIC targets in dogs.

Dosing Interval	%Time above MIC	Attainment (%)		
		MRSP	MSSP	*p*-Value
q 24 h	33.33	0.00	0.00	>0.9999
q 12 h	66.66	43.75	52.27	0.7710
q 8 h	100.00	43.75	52.27	0.7710

MRSP; Methicillin-resistant *S. pseudintermedius*. MSSP; Methicillin-susceptible *S. pseudintermedius*. q; every (quaque).

## Data Availability

The data supporting this study are available from the corresponding author upon reasonable request.
